# Effect of simple vs. extended cholecystectomy on prognosis of T1b gallbladder cancer: a systematic review and meta-analysis

**DOI:** 10.3389/fsurg.2025.1477301

**Published:** 2025-05-26

**Authors:** Hongpeng Gu, Guoqiang Zhang, Haijie Ma, Ze Yu

**Affiliations:** ^1^ Department of Colorectal Surgery, Zhejiang Hospital of Integrated Traditional Chinese and Western Medicine, Hangzhou, Zhejiang, China; ^2^ Department of General Surgery, Zhoushan Hospital, Wenzhou Medical University, Zhoushan, Zhejiang, China; ^3^ The Laboratory of Cytobiology and Molecular Biology, Zhoushan Hospital, Wenzhou Medical University, Zhoushan, Zhejiang, China

**Keywords:** gallbladder cancer, simple cholecystectomy, extended cholecystectomy, prognosis, meta-analysis

## Abstract

**Background & aims:**

Extended cholecystectomy (EC) is recommended for T1b gallbladder cancer (GBC), but the optimal surgical procedure for T1b GBC remains controversial. This study aims to compare the prognosis of T1b GBC patients who underwent simple cholecystectomy (SC) vs. EC from a long-term survival perspective.

**Methods:**

We performed a systematic search up to August 06, 2024, using MEDLINE (PubMed), EMBASE, Web of Science and Cochrane Library. The main outcomes were overall survival (OS) and disease-specific survival (DSS). We evaluated the quality of the studies included and the risk of bias, calculated the pooled hazard ratios (HRs) for OS and DSS and conducted the sensitivity analysis.

**Results:**

A total of 8 retrospective studies involving 2,097 T1b GBC patients (SC = 1,263, EC = 408) were included. The pooled result of OS showed that the EC group had a significantly better OS than the SC group (pooled HR = 0.73; 95% CI = 0.59–0.89; *P* = 0.002). The pooled result of DSS indicated that EC significantly improved DSS of T1b GBC compared to SC (pooled HR = 0.47; 95% CI = 0.29–0.77; *P* = 0.003).

**Conclusions:**

EC should be chosen as the optimal surgical procedure for patients with T1b GBC from the standpoint of long-term postoperative survival. However, further analysis of more comprehensive studies will be necessary in the future to improve the quality of evidence.

**Systematic Review Registration:**

https://www.crd.york.ac.uk/PROSPERO/view/CRD42023449431, PROSPERO CRD42023449431.

## Introduction

Gallbladder cancer (GBC) is the most common malignant tumor of the biliary system with high lethality (1). Although the incidence of GBC is relatively low, with 115,949 new cases in 2020, ranking 25th among all 36 common tumors, the prognosis of GBC is poor with a five-year survival rate of less than 5% for advanced GBC, since the patients are always diagnosed at an advanced stage (2–4). Treatment of GBC includes surgery and adjuvant therapy, with surgery being the primary method of curing GBC, while the value of adjuvant therapy remains unclear (5–9). In terms of surgery, R0 resection (negative surgical margins) is considered as an important factor affecting the long-term prognosis of GBC, which has been a consensus stated by experts (10–12). The choice of surgical procedure mainly depends on the stage of GBC, however, the extent of R0 resection for T1 GBC has been controversial, especially for T1b GB (1, 13).

According to the eighth edition of the American Joint Committee on Cancer (AJCC) staging criteria, T1 GBC includes T1a and T1b GBC, which represents tumor invasion of the lamina propria and the muscular layer, respectively (14). The main controversy over the surgical strategy for T1 GBC has always been whether extended cholecystectomy (EC) is necessary or whether simple cholecystectomy (SC) is adequate (15, 16). SC indicates removal of the gallbladder only, and EC represents removal of the adjacent liver tissue (wedge resection or IVb and V hepatic segmental resection) with regional lymph node dissection in addition to removal of gallbladder (1, 14). Based on the high five-year cumulative survival rate of T1a GBC after SC, which is above 95%, SC alone as a treatment for T1a GBC is generally considered reasonable (12, 17–19). As for T1b GBC, the National Comprehensive Cancer Network (NCCN) guidelines and the Clinical practice guidelines of Japan recommend that T1b or greater lesions should receive radical surgery (20, 21). However, SC still accounts for a significant portion of the surgical volume for T1b GBC in clinical practice, which means that, as a recommended procedure for T1b GBC, EC is still under- recognized (22–24). In addition, several studies have concluded EC does not significantly improve the long-term prognosis of patients with T1b GBC compared to SC (23, 25). One study even noted that there is a trend toward worse survival in T1b GBC receiving EC, though no statistically significant difference is shown (26).

There was a systematic review and meta-analysis about this controversy, which concluded that SC and EC show no difference in patients with T1b GBC in terms of long-term survival. However, the authors of the study argued that the conclusions are limited by a number of factors, including insufficient survival data and lack of standardization of surgical methodology and pathology reports (16). After first meta-analysis was published, a number of higher-quality articles with more comprehensive data have been published in response to the controversy. Therefore, we collected related studies published up to the year 2024 and conducted an updated systematic review and meta-analysis to reevaluate which surgical procedure is better for T1b GBC from a long-term survival perspective.

## Methods

The protocol of this research was registered in advance in the International Prospective Register of Systematic Reviews. (CRD42023449431). This meta-analysis was conducted following the Preferred Reporting Items for Systematic Reviews and Meta-Analyses (PRISMA) guidelines (27).

### Data sources and search strategy

The literature search was performed using MEDLINE (PubMed), EMBASE, Web of Science and Cochrane Library, up to August 06, 2024, with language restricted to English. The literatures were retrieved by combining medical subject headings (MeSH) terms with entry terms. The details of search terms and search strategy are shown in Table 1.

**Table 1:** Detailed search strategy according to database.

PubMed Search Strategy (211 results) ((((((((((((((((((“Gallbladder Neoplasms"[Mesh]) OR (Gallbladder Neoplasm)) OR (Neoplasm, Gallbladder)) OR (Neoplasms, Gallbladder)) OR (Cancer of Gallbladder)) OR (Gallbladder Cancers)) OR (Gallbladder Cancer)) OR (Cancer, Gallbladder)) OR (Cancers, Gallbladder)) OR (Gall Bladder Cancer)) OR (Bladder Cancer, Gall)) OR (Bladder Cancers, Gall)) OR (Cancer, Gall Bladder)) OR (Cancers, Gall Bladder)) OR (Gall Bladder Cancers)) OR (Cancer of the Gallbladder)) AND ((((((T1) OR (T1b)) OR (Nevin)) OR (Nevin II)) OR (Muscularis)) OR (Muscular))) AND ((“Cholecystectomy"[Mesh]) OR (Cholecystectomies))) AND ((survival) OR (mortality))
Embase Search Strategy (325 results) #1 ‘gallbladder tumor'/syn #2 ‘t1’ OR ‘t1b’ OR ‘nevin'/exp OR ‘nevin’ OR ‘nevin ii’ OR ‘muscularis’/exp OR ‘muscularis’ OR ‘muscular’ #3 ‘cholecystectomy'/exp OR ‘cholecystectomy’ OR ‘cholecystectomies’ #4 ’survival'/exp OR ‘survival’ OR ‘mortality'/exp OR ‘mortality’ #5 #1 AND #2 AND #3 AND #4
Web of Science Search Strategy (265 results) #1 Gallbladder Neoplasms (Topic) or Gallbladder Neoplasm (Topic) or Neoplasm, Gallbladder (Topic) or Neoplasms, Gallbladder (Topic) or Cancer of Gallbladder (Topic) or Gallbladder Cancers (Topic) or Gallbladder Cancer (Topic) or Cancer, Gallbladder (Topic) or Cancers, Gallbladder (Topic) or Gall Bladder Cancer (Topic) or Bladder Cancer, Gall (Topic) or Bladder Cancers, Gall (Topic) or Cancer, Gall Bladder (Topic) or Cancers, Gall Bladder (Topic) or Gall Bladder Cancers (Topic) or Cancer of the Gallbladder (Topic) #2 T1 (Topic) or T1b (Topic) or Nevin (Topic) or Nevin II (Topic) or Muscularis (Topic) or Muscular (Topic) #3 Cholecystectomy (Topic) or Cholecystectomies (Topic) #4 survival (Topic) or mortality (Topic) #5 #1 AND #2 AND #3 AND #4
Cochrane Library Search Strategy (8 results) #1 MeSH descriptor: [Gallbladder Neoplasms] explode all trees #2 (Gallbladder Neoplasms or Gallbladder Neoplasm or Neoplasm, Gallbladder or Neoplasms, Gallbladder or Cancer of Gallbladder or Gallbladder Cancers or Gallbladder Cancer or Cancer, Gallbladder or Cancers, Gallbladder or Gall Bladder Cancer or Bladder Cancer, Gall or Bladder Cancers, Gall or Cancer, Gall Bladder or Cancers, Gall Bladder or Gall Bladder Cancers or Cancer of the Gallbladder):ti,ab,kw (Word variations have been searched) #3 #1 or #2 #4 (T1 or T1b or Nevin or Nevin II or Muscularis or Muscular):ti,ab,kw #5 MeSH descriptor: [Cholecystectomy] explode all trees #6 (Cholecystectomies):ti,ab,kw (Word variations have been searched) #7 #5 or #6 #8 (survival or mortality):ti,ab,kw #9 #3 and #4 and #7 and #8

### Study selection

Two authors (G.H.P., Y.Z.) independently reviewed the titles, abstracts, and the full-text was evaluated when necessary. Disagreements were resolved by consulting a third team member (M.H.J.).

The inclusion criteria were as follows: (1) population: Patients with a final pathologic diagnosis of T1b GBC (gallbladder adenocarcinoma), whether it is GBC that has been clarified preoperatively by imaging or incidental GBC found by postoperative pathology; (2) intervention: the studies including the comparison of SC and EC; (3) study design: randomized controlled trials (RCTs) or cohort studies; (4) outcomes: sufficient data describing overall survival (OS) or disease-specific survival (DSS) with follow-up longer than 5 years.

The following studies were excluded: (1) reviews, case reports, letters, conference abstracts or guidelines; (2) studies with experimental or control group sample size of less than five; (3) when data were overlapped from the same database with same study periods, studies with insufficient data were excluded; (4) studies without hazard ratios (HRs) or Kaplan–Meier (KM) survival curves comparing the two surgical strategies. We defined the SC as cholecystectomy alone without lymphadenectomy, and the EC was identified as cholecystectomy including a wedge resection of the gallbladder bed in the liver or segmentectomy of liver segments IVb and V with regional lymphadenectomy regardless of whether open or laparoscopic, and whether extended surgery in the first procedure or secondary enlargement for incidental GBC. Therefore, studies that did not meet the definition of these two surgical strategies were also excluded.

### Data extraction and quality assessment

The main outcomes were OS and DSS. Data were extracted by two authors (G.H.P., Y.Z.) independently according to the predefined data collection form. The following data were extracted from included studies: first author, publication year, study period, country of included patients, study design, median follow-up, sample size, gender, age, 5-year OS rate, 5-year DSS rate, HR of OS and HR of DSS. For studies with only KM survival curves but no HRs, data were extracted from KM survival curves using Engauge Digitizer11.1, and HRs were estimated following the methods provided by Tierney et al. (28).

The quality of included studies was assessed using the Newcastle-Ottawa Scale (NOS) by two authors (G.H.P., Y.Z.) independently (29). Studies with a quality score ≤4 were considered low-quality studies and would be excluded. Disagreements were resolved by consulting a third team member (M.H.J.) during data extraction and quality assessment.

### Data synthesis and statistical analysis

The pooled HR with 95% confidence interval (CI) was used to explore risk factors for OS and DSS of T1b GBC. Statistical heterogeneity was assessed using Q statistical test and I2 statistical test. Fixed-effects model was chosen when there was no significant heterogeneity (Q tests, *P* > 0.1; I2 < 50%), otherwise, random-effects model was used.

Sensitivity analysis was conducted to assess the stability and reliability of the pooled results of the meta-analysis by eliminating eligible studies one by one. Publication bias was assessed using funnel plots and the Egger's test.

Statistical analysis was performed using the Review Manager (RevMan) (version 5.3; The Cochrane Collaboration, The Nordic Cochrane Center, Copenhagen, Denmark) and Stata software Version 12.0 (Stata Corp. LP, College Station, Texas, USA). Statistical significance was defined as a two-tailed *P* value less than 0.05.

## Results

### Systematic search and characteristics

**Figure 1:**
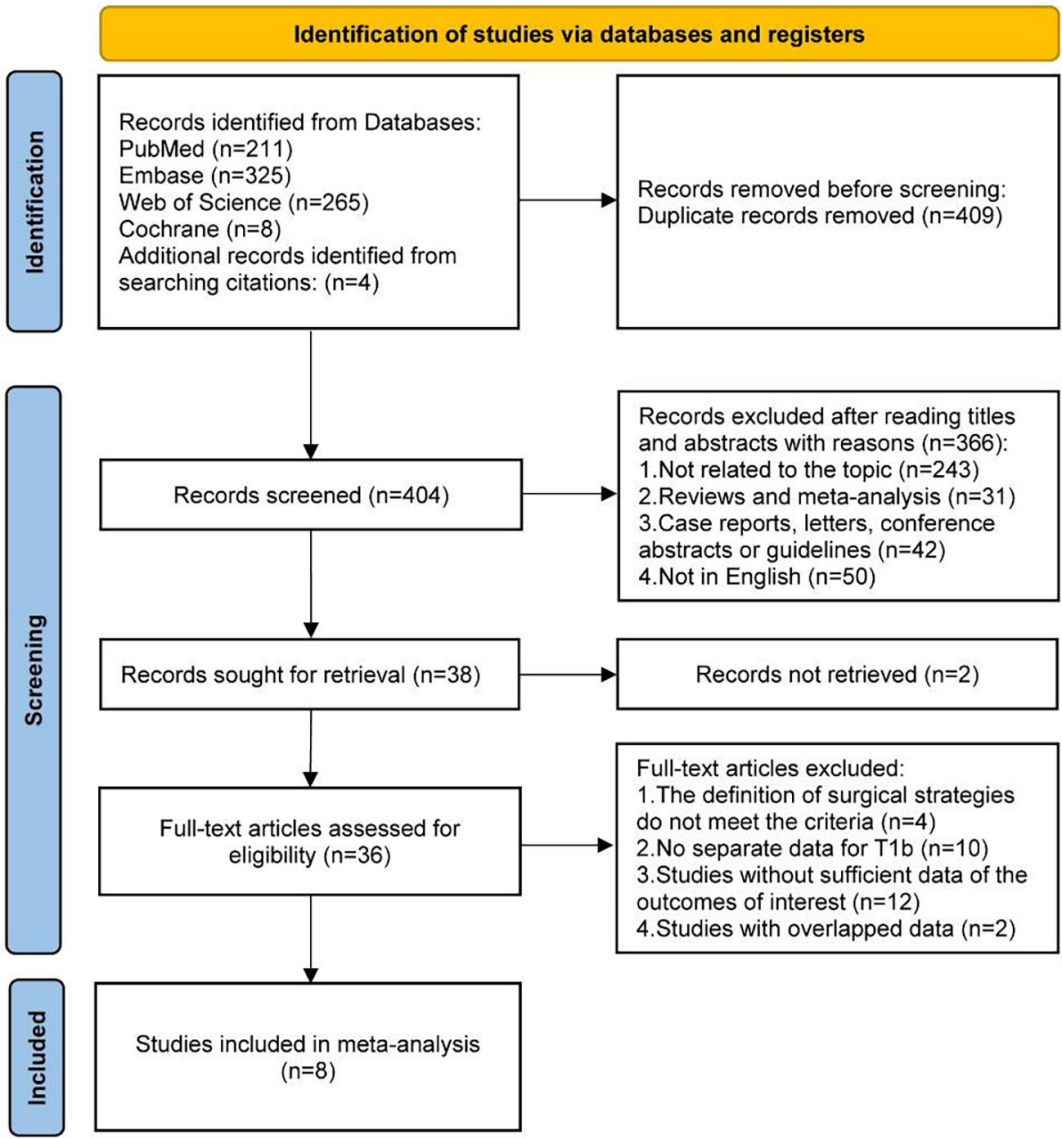
PRISMA flow diagram of the studies included in the meta-analysis.

After an initial systematic search, a total of 813 studies were identified, of which 409 duplicate records were excluded. 366 studies were further excluded by reading the titles and abstracts, and 2 studies were also excluded for records not retrieved. The 36 remaining studies were assessed for eligibility through full-text reading, and 8 studies were included in the final analysis (Figure 1) (22, 24–26, 30–33).

The characteristics of included studies are described in Table 2. The 8 included studies were all retrospective cohort studies and included a total of 2,097 patients with T1b GBC, of which 1,263 patients received SC and 408 patients received EC, with one study not describing the exact number of patients who received SC or EC (26). The lowest quality score was 6 among all studies and the details of quality assessment of all included studies are presented in Table 3.

**Table 2:** Characteristics of the include studies.

Study	Study period	Country of included patients	Study design	Median follow-up（months）	Number of patients			Male(%)		Age(years)		5-year OS rate(%)		5-year DSS rate(%)		NOS score
					Total	SC	EC	SC	EC	SC	EC	SC	EC	SC	EC	
Downing et al. (26)	1988-2005	USA	Retrospective	33	426	Nr	Nr	Nr	Nr	Nr	Nr	Nr	Nr	Nr	Nr	6
Goetze et al. (30)	1997-2007	Germany	Retrospective	Nr	72	49	23	Nr	Nr	Nr	Nr	42	79	Nr	Nr	6
Hari et al. (31)	1988-2008	USA	Retrospective	22 (7-244)^c^	457	427	30	Nr	Nr	Nr	Nr	37.03	54.71	51.62	90.86	7
Lee et al. (32)	1995-2004	Korea	Retrospective	60.8 (1.1– 174.5)^a^	141	89	52	Nr	Nr	Nr	Nr	Nr	Nr	86.84	92.7	6
Shao et al. (22)	2000-2017	USA	Retrospective	Nr	397	346	51	Nr	Nr	Nr	Nr	Nr	Nr	Nr	Nr	8
Tashiro et al. (33)	1960-1978	Japan	Retrospective	Nr	86	69	17	Nr	Nr	Nr	Nr	58.1	60	Nr	Nr	6
Vo et al. (24)	2004–2012	USA	Retrospective	Nr	464	247	217	31.98	35.02	66.2 ± 10.5^b^	64.4 ± 10.4^b^	48.3	57.5	Nr	Nr	8
Yoon et al. (25)	1997-2010	Korea	Retrospective	96.8 (17–215)^a^	54	36	18	47.22	55.56	61.6 (35–79)^a^	56.6 (40–70)^a^	88.8	93.3	Nr	Nr	9

DSS, disease-specific survival; EC, extended cholecystectomy; Nr, not reported; OS, overall survival; SC, simple cholecystectomy.

^a^ Mean(range).

^b^ Mean ± SD.

^c^ Median (range).

**Table 3:** Quality assessment of the include studies.

Study	A	B	C	D	E	F	G	H	Quality score
Downing et al. (26)	1	1	1	1	0	1	1	0	6
Goetze et al. (30)	1	1	1	1	0	1	1	0	6
Hari et al. (31)	1	1	1	1	1	1	1	0	7
Lee et al. (32)	1	1	1	1	0	1	1	0	6
Shao et al. (22)	1	1	1	1	2	1	1	0	8
Tashiro et al. (33)	1	1	1	1	0	1	1	0	6
Vo et al. (24)	1	1	1	1	2	1	1	0	8
Yoon et al. (25)	1	1	1	1	2	1	1	1	9

NOS analysis criteria: A: Representativeness of the exposed cohort; B: Selection of the non-exposed cohort; C: Ascertainment of exposure; D: Demonstration that outcome of interest was not present at start of study; E: Comparability of cohorts on the basis of the design or analysis; F: Assessment of outcome; G: Was follow-up long enough for outcomes to occur; and H: Adequacy of follow up of cohorts.

### OS

**Figure 2:**
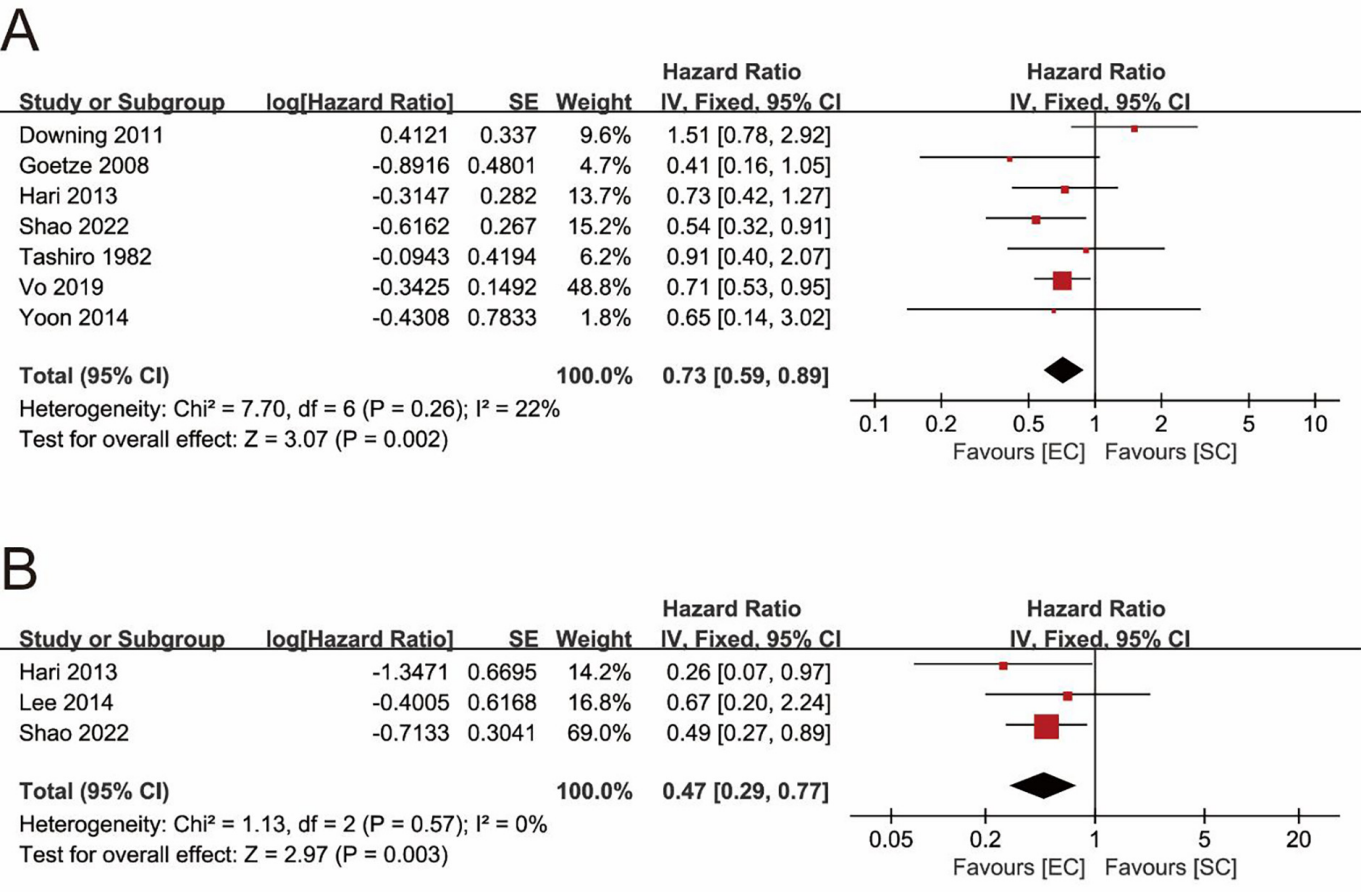
Forest plots of main outcomes. **(A)** Overall survival. **(B)** Disease-specific survival.

**Figure 3:**
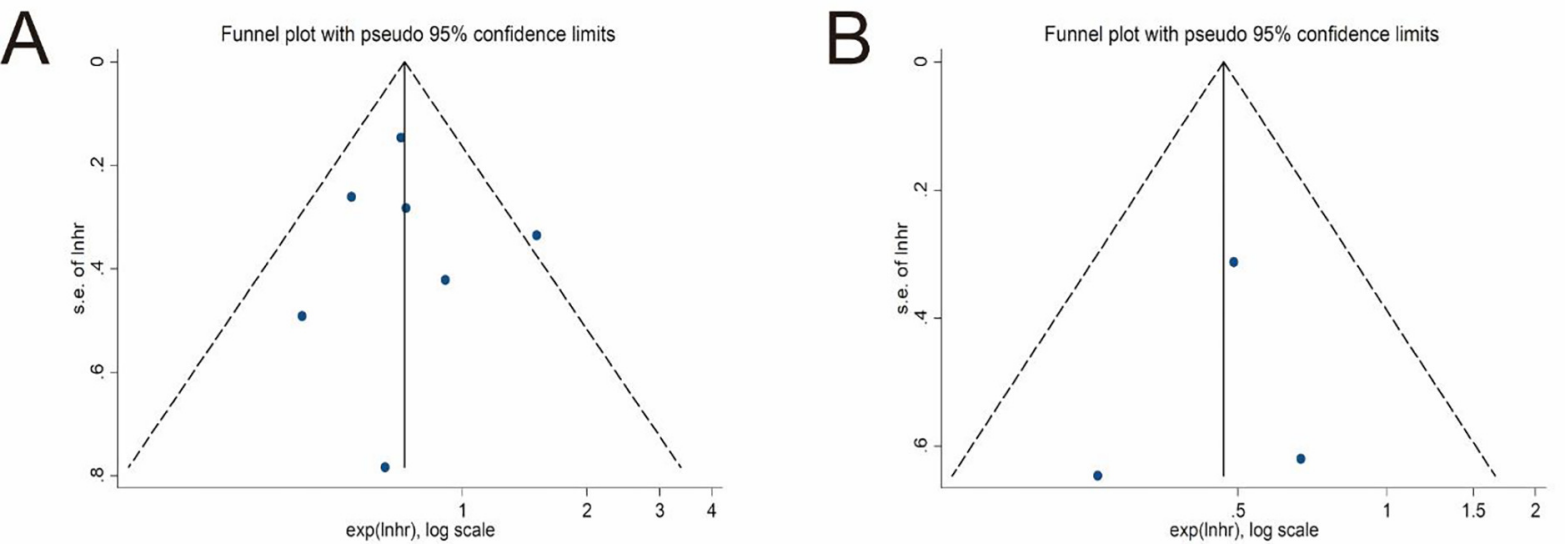
Funnel plots of main outcomes. **(A)** Overall survival. **(B)** Disease-specific survival.

Seven studies explored the impact of surgical procedures (SC vs. EC) on the long-term survival of T1b GBC by providing HR values or KM survival curves for OS (22, 24–26, 30, 31, 33). The pooled result showed that the EC group had a significantly better OS (pooled HR = 0.73; 95% CI = 0.59- 0.89; *P* = 0.002) (Figure 2A). No significant heterogeneity (*I^2^* = 22%, *P* = 0.26) and statistical (Egger's test, *P* = 0.915) or visual (Figure 3A) evidence of publication bias were observed.

### DSS

Only 3 studies compared the differences in DSS between the SC and EC groups (22, 31, 32). The pooled result indicated that EC significantly improved DSS of T1b GBC compared to SC (pooled HR = 0.47; 95% CI = 0.29–0.77; *P* = 0.003) (Figure 2B) without significant heterogeneity (*I^2^* = 0%, *P* = 0.57) and statistical (Egger's test, *P* = 0.816) or visual (Figure 3B) evidence of publication bias.

### Sensitivity analysis

**Figure 4:**
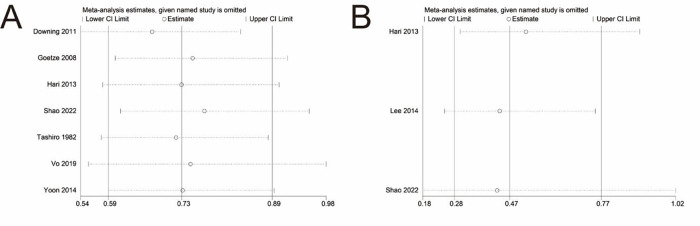
Sensitivity analysis of main outcomes. **(A)** Overall survival. **(B)** Disease-specific survival.

Sensitivity analyses were conducted on the pooled results of OS and DSS. The pooled result of OS did not change after eliminating any individual study, which confirmed the stability and reliability of the pool result (Figure 4A). However, when the study reported by Shao et al. was excluded, the significance of the pooled result of DSS disappeared (Figure 4B), suggesting that the pooled result of DSS was not robust and greatly influenced by the data reported by Shao et al. (22).

## Discussion

This systematic review and meta-analysis of 8 retrospective cohort studies provided an overview of the evidence comparing different surgical procedures on the long-term survival of T1b GBC. Our study found that EC led to better long-term survival outcomes for T1b GBC compared to SC. From an OS perspective, EC reduced the risk of outcome events in T1b GBC by 27% and reduced the risk of outcome events by 53% in terms of DSS.

The recurrence and lymph node metastasis rates of GBC are as high as approximately 50%, meanwhile, the deeper the invasion of GBC, the higher the degree of malignancy and the higher the risk of metastasis (34–36). From a more specific perspective, the incidence of residual or metastatic lesions after SC for GBC is similarly high (11, 37). Moreover, GBC spreads early through lymph, blood, or direct infiltration into the liver. Although T1b GBC does not penetrate the entire gallbladder wall and cannot directly infiltrate the liver, it may produce undetectable micrometastases through lymph and venous blood. Anatomically, cholecystic venous blood most frequently enters the peripheral portal vein branches of hepatic segment IV and V (38), and there are small veins in the connective tissue between the gallbladder and liver that directly enter the liver parenchyma (39, 40), through which GBC cells can metastasize to the liver parenchyma of the IVb and V segments surrounding the gallbladder and form local intrahepatic metastases. Consequently, as opposed to SC, EC removes the liver tissue at high risk for GBC metastasis, which are the liver tissue adjacent to the gallbladder bed and the segment of the liver with the most frequent inflow of the gallbladder vein, and the regional lymph nodes, which means the potential micro-metastatic lesions are removed as well. It may be in this way that EC reduces the postoperative metastasis or recurrence of T1b GBC, thereby significantly improving their long-term survival outcomes. Meanwhile, the conclusion of our study suggested that EC was more effective in reducing the risk of outcome events of DSS than reducing the risk of outcome events of OS in T1b GBC (53% vs. 27%), which laterally verified that EC might play a role by improving the postoperative metastasis or recurrence in T1b GBC. However, due to the lack of data related to recurrence or metastasis in most of the studies included in this meta-analysis, it is not possible to validate the above speculation by data analysis for the time being.

The diagnosis of T1b GBC can be broadly categorized into two types, T1b GBC clinically diagnosed with the aid of imaging, namely cT1b GBC, and pT1b incidental GBC, which is definitively diagnosed by intraoperative or postoperative pathology. In fact, close to half of late GBC and two-thirds of early GBC are diagnosed incidentally (not suspected before or during surgery) and are usually concealed by mucosal changes caused by acute cholecystitis during routine examination of the gallbladder (1)**.** It is in view of this situation that both cT1b GBC and pT1b incidental GBC were included in our study. Therefore, we did not specifically differentiate between the above two cases during the search, but rather targeted GBC with a final pathologic diagnosis of T1b. Consequently, when we searched for extended cholecystectomy, we included the literature based on the scope of the procedure only, including both the first surgery with extended cholecystectomy and the second extended surgery due to a diagnosis of pT1b incidental GBC after simple cholecystectomy. For this reason, the results of our study can be interpreted as follows: for cT1b GBC, extended cholecystectomy is the appropriate surgical procedure, whereas for pT1b incidental GBC, given the risk of residual lesions in the gallbladder fossa or regional lymph nodes, a second extended procedure is usually recommended, including removal of the adjacent liver tissue (wedge resection or IVb and V hepatic segmental resection) with regional lymph node dissection.

A previous systematic review and meta-analyses have reported that SC is comparable to EC with regard to overall survival in T1b GBC (16), but this study was limited by the number of cases included in the study and the lack of standardization of key factors. Our study excluded the studies with less than 5 samples in the experimental or control group included in the previous study and included newly published research on this topic. Meanwhile, we also standardized the definition of extent of surgical procedures. In addition, we included DSS as a new outcome indicator and used HR as a new effect value, which better and more comprehensively reflected the risk profile throughout the postoperative period of T1b GBC by excluding time factors. Therefore, despite the inconsistency with previous findings, our conclusions are more current and can be used as additional evidence to the mainstream guidelines as well as consensus.

However, there are some limitations that should be acknowledged in our study. First, the unstable results of sensitivity analysis on DSS may be attributed to insufficient studies included in the analysis. Second, the HRs estimated from KM survival curves were the result of log-rank tests that did not exclude the effect of confounding factors, and the process of extracting data from the KM survival curves caused slight errors as well. Third, our study only considered the long-term survival outcomes of patients and did not evaluate the impact of SC and EC on T1b GBC in other ways, such as postoperative complications, quality of life, and so on. Fourth, the influence of the type of surgical approach, whether open or laparoscopic, was not considered in our study. Finally, the eight studies included in our analysis are all retrospective cohort studies, with five studies having moderate quality, therefore the evidence level of these studies is relatively low.

Robotic surgery in gallbladder cancer has demonstrated an advantage in the precision of lymph node dissection in key areas such as hilar liver and peripancreatic head. For example, a case of single-hole robotic surgery showed that using the flexibility of the robotic arm and the high-definition three-dimensional field of view, the 7th, 8th, 12th, and 13th lymph nodes required for gallbladder cancer could be completely removed, and the postoperative pathologically confirmed negative margin rate was as high as 91% (41). Compared with traditional laparoscopy, the robotic system reduces the problem of limited field of view due to instrument conflict (42). Robotic surgery has demonstrated technical feasibility and short-term efficacy advantages in the treatment of gallbladder cancer, but its full promotion needs to address key issues such as device suitability, cost-effectiveness and insufficient long-term oncologic evidence. In the next 5–10 years, as technology iterations and high-quality evidence accumulate, robotic surgery is expected to become an important option for minimally invasive treatment of early gallbladder cancer.

## Conclusions

The findings of our research supported that EC should be chosen as the optimal surgical procedure for patients with T1b GBC from the standpoint of long-term postoperative survival. Specifically, for cT1b GBC, extended cholecystectomy is the appropriate surgical procedure, whereas for pT1b incidental GBC, a second extended procedure is usually recommended. However, more future studies with large-scale and more comprehensive data are essential to strengthen the current findings and guide the clinical treatment through an evidence-based approach.

## Data Availability

The original contributions presented in the study are included in the article/Supplementary Material, further inquiries can be directed to the corresponding authors.
